# Revisiting the use of graph centrality models in biological pathway analysis

**DOI:** 10.1186/s13040-020-00214-x

**Published:** 2020-06-16

**Authors:** Pourya Naderi Yeganeh, Chrsitine Richardson, Erik Saule, Ann Loraine, M. Taghi Mostafavi

**Affiliations:** 1 Beth Israel Deaconess Medical Center, Harvard Medical School, 330 Brookline Ave., Boston, 02215 MA USA; 2 Department of Computer Science, The University of North Carolina at Charlotte, 9201 University City Blvd, Charlotte, 28223 NC USA; 3 Department of Biological Sciences, The University of North Carolina at Charlotte, 9201 University City Blvd, Charlotte, 28223 NC USA; 4 Department of Bioinformatics and Genomics, The University of North Carolina at Charlotte, 9201 University City Blvd, Charlotte, 28223 NC USA

**Keywords:** Biological networks, Network analysis, Pathway analysis

## Abstract

The use of graph theory models is widespread in biological pathway analyses as it is often desired to evaluate the position of genes and proteins in their interaction networks of the biological systems. In this article, we argue that the common standard graph centrality measures do not sufficiently capture the informative topological organizations of the pathways, and thus, limit the biological inference. While key pathway elements may appear both upstream and downstream in pathways, standard directed graph centralities attribute significant topological importance to the upstream elements and evaluate the downstream elements as having no importance.We present a directed graph framework, Source/Sink Centrality (SSC), to address the limitations of standard models. SSC separately measures the importance of a node in the upstream and the downstream of a pathway, as a sender and a receiver of biological signals, and combines the two terms for evaluating the centrality. To validate SSC, we evaluate the topological position of known human cancer genes and mouse lethal genes in their respective KEGG annotated pathways and show that SSC-derived centralities provide an effective framework for associating higher positional importance to the genes with higher importance from a priori knowledge. While the presented work challenges some of the modeling assumptions in the common pathway analyses, it provides a straight-forward methodology to extend the existing models. The SSC extensions can result in more informative topological description of pathways, and thus, more informative biological inference.

## Introduction

Biological pathways represent sets of bio-molecular entities, such as genes and proteins, and their cascades of interactions which associate with certain cellular functions [1]. The abundance and availability of annotated pathways is a key element in bridging the gap between molecular level dynamics and high-level biological insight [2–5]. Although changes in individual molecules may trigger variations in the cellular programs, many biological functions emerge from the systematic behaviour of entities and interactions. This systems biology concept positions the use of pathways in a significant value for discovery, treatment, diagnosis, and prediction in biomedical studies [6–9].

The term “Pathway Analyses” describes a category of models that leverage biological interaction networks for the study of molecular-level data, such as gene expression. Many of these tools are built on the premise of a well-established body of literature which indicates that the position of genes/proteins in their associated interaction networks can determine their importance in biological systems of interest [10–12]. For example, several network-based pathway enrichment analysis models (N-PEM) use graph theory concepts to prioritize topologically important differential expressions in the pathways and produce functional interpretations [13–21].

Graph centrality models are the premier methods for evaluating the topological positions of individual network entities [22]. While these models have been successfully utilized in pathway analyses for functional interpretation, their abstractions of network organizations do not necessarily capture key topological features of pathways, suggesting a potential for a more biologically relevant assessment of pathways. Biological pathways, particularly signaling pathways, appear as an upstream-to-downstream organization, indicating a temporal and biochemical order of interactions between associated genes and proteins. In a directed graph model, upstream pathway elements are mostly represented as nodes with no incoming edges and downstream elements are represented as nodes with no out-going edges. Subsequently, standard centrality model for directed graphs, such as PageRank and Katz, do not assign any topological importance of the downstream elements, many of which have been shown to be key elements of biological functions.

The goal of this study is to quantitatively show the limitation of the standard centrality models and provide a plausible alternative to improve the utility of topological evaluations of pathways. We hypothesize that a directed centrality model which accounts for the topological position of key elements at downstream and upstream ends of pathways can provide a more meaningful characterization of biological networks. To achieve our goal, we first formalize the standard centrality models into three categories of Source, Sink, and undirected frameworks. The Source framework indicates a version of centrality models that can capture the importance of a node as a sender of information, which relates to the directed graph models used in typical pathway analyses. The Sink framework aims to capture to identify important receivers of biological information/signals. We then introduce Source/Sink centrality (SSC) concept, which is a flexible framework that works by applying any centrality model to a graph and its transposed graph simultaneously, and combining the two resulting profiles. SSC produces a centrality score for each node in a network that quantifies the importance of each gene both upstream and downstream of a pathway while accounting for the order and the direction of the interactions.

In a recent preliminary study, we reported that the SSC framework of common centrality models provides a more informative characterization of key pathway elements’ positions in contrast to the standard directed models [23]. In particular, we showed that the centrality scores produced by SSC have a stronger correlation and more descriptive linear relationship with the probability of a gene to be important based on a priori known biological functions. In another study, we showed an application of SSC modification of Katz centrality in network-based enrichment analysis to prioritize the differential expression topologically important genes. In that case, we showed that the SSC framework produces a more biologically relevant functional interpretation of disease genomic data [24]. Following that in a recent study, Zaffaroni et al. leveraged the SSC modification of Katz centrality for predicting the driver pathways of cellular transition [25, 26].

In this study, we expand SSC modeling to multiple spectral centrality models and validate it using additional and updated background data. In particular, we investigate a battery of standard graph centrality models and their SSC extensions for describing the organization of a priori known important genes. For a priori important genes, we focus on human cancer genes and mouse lethal genes, also known as essential genes, i.e. genes whose knockdown results in the death of organisms. The rationale for choosing the cancer-related genes is the intuition that cancers are regarded as diseases of pathways, i.e. cancers are primarily driven by perturbation/alteration of pathways [7, 27]. Subsequently, the dysfunction of one or more cancer-related genes can result in dysfunction of their associated pathways [7]. Therefore, understanding the topological position of cancer associated genes may reveal insight regarding the topological organization of key pathway drivers/regulators. The rationale for choosing mouse lethal genes is the existence of an extensive literature on the relationship between centrality and lethality in protein-protein interaction networks, where it has been shown that higher centrality correlates with higher probability of being lethal (essential) [28–30].

From multiple perspectives, we show that the SSC extensions, in comparison to the standard models, produce a more descriptive topological framework for the positions of cancer gene in human pathways, as well as that of essential genes in mouse pathways. These results show that the SSC methodology contributes to the biological pathway analyses and inference methods by providing a more realistic framework for measuring network positions.

## Material and methods

### Graph modeling of pathways

Let a directed graph, *G*=(*V*,*E*), represent a pathway where $V(G) = \left \{v_{1},v_{2},\dots,v_{n}\right \}$ is the set of nodes and $E(G) = \left \{e_{1},e_{2},\dots, e_{m} \right \}$ is the set of edges. Each edge, *e*_*k*_=(*v*_*i*_,*v*_*j*_), is an ordered pair that indicates a directed relationship from gene-encoded element *v*_*i*_ to *v*_*j*_. A graph can be alternatively represented as an undirected graph where the edges are unordered pairs.

For any graph, the *neighborhood* of a node *v*_*i*_, *N*(*v*_*i*_), is the set of all adjacent nodes of *v*_*i*_, *N*_*G*_(*v*_*i*_)={*v*_*j*_|(*v*_*i*_,*v*_*j*_)∈*E*(*G*)}. The *degree* of a node is defined as the size of its neighborhood, *deg*(*v*_*i*_)=|*N*_*G*_(*v*_*i*_)|. For a directed graph, the former notion of degree is referred to as *out-degree*, *deg*^+^(*v*). For a directed graph, neighborhood and degree can be also defined based on in-coming edges, i.e. *in-degree*, *deg*^−^(*v*_*i*_)=|{*v*_*j*_∣(*v*_*j*_,*v*_*i*_)∈*E*}|. A graph with *n* vertices has an equivalent representation of a *n*×*nadjacency matrix*, *A*_*G*_. Formally:

1 $$ [A_{G}]_{ij}= \left\{\begin{array}{cc} 1, & \left(v_{i},v_{j}\right) \in E \\ 0, & \text{otherwise} \end{array}\right.  $$

The *transpose* of a graph, *G*^*T*^, is a graph with reversed edge directions, where *V*(*G*^*T*^)=*V*(*G*) and *E*(*G*^*T*^)={(*u*,*v*)|(*v*,*u*)∈*E*(*G*)}, thus $A_{G^{T}} = A_{G}^{T}$. A graph *centrality* is a function, *C*(*v*), $C : V(G) \rightarrow \mathbb {R}$, for describing a topological scoring (importance) of the nodes in a network [22].

*Degree Centrality* of each node is the size of its neighborhood. Studies have shown that the degree of nodes in protein-protein interaction networks of different organisms correlates with their essentiality, meaning the likelihood of a protein’s removal, e.g. knockdown, to be lethal for the model organism [10, 29, 30]. Here, we calculated degree centrality as the sum of in-degree and out-degree, which the same as the degree centrality in the underlying undirected graph:

2 $$ C_{deg}(v) = deg^{+}(v) + deg^{-}(v)   $$

*PageRank Centrality* is a spectral centrality measure where the importance of a node is a function of the centrality of its neighbors. In its original definition, PageRank describes the probability distribution of a uniform random walk with restart being present at each node of a graph after a large number of steps [22, 31, 32]. In graph theory terms, the PageRank of a node *v* is based on the PageRank of the nodes with links to *v*, divided by their out degrees. Formally:

3 $$ C_{pgr}(v_{i}) =\beta_{i} + \alpha\sum\limits_{v_{j} | v_{i} \in {N_{G}(v_{j})}} \frac{C_{pgr}(v_{j})}{|{N_{G}(v_{j})}|}   $$

*β*_*i*_’s are constant values that relate the probability of restarting at node *v*_*i*_. The parameter *α* is a dampening factor that relates to the transition probability of the random walk. The Formula 3 can be expressed in a vectorized format as following:

4 $$ C_{pgr} = \beta + \alpha A^{T}D^{-1} C_{pgr}   $$

where *C*_*pgr*_ is the vector of centralities and *β* is the vector of initial values. *D* is the diagonal (out) degree matrix such that [*D*]_*ii*_=*max*(*deg*^(+)^(*v*_*i*_),1). A closed form solution of Formula 3 is achieved by solving for *C*_*pgr*_ [22]. Formally:

5 $$ C_{pgr} = \left(I- \alpha A^{T}D^{-1}\right)^{-1}\beta   $$

PageRank can be used for both directed and undirected graphs. Closely related notions of PageRank have been used in applications of pathway analysis [14, 17].

We define the *PageRank Sink* centrality as the standard PageRank of a directed graph. The original concept of PageRank, as described by Brin and Page, measures the importance of a website based on the importance of the websites that have a link to it [31]. Likewise, in the Sink component of the PageRank, the downstream nodes have the higher importance. This is because a random walk will not be present at any node without incoming edges, unless by a restart event. The PageRank Sink centrality captures the importance of a node as a receiver of information. Formally we define the Sink PageRank centrality ($C_{pgr}^{Si}$) as:

6 $$ C_{pgr}^{Si}(v) := C_{pgr}(v)  $$

To modify PageRank in such a way that captures the importance of nodes as source of signal, we derive a PageRank score when applied to the transpose of a graph. Formally, we define the *PageRank Source*$\left (C_{pgr}^{So}\right)$ as:

7 $$ C_{pgr}^{So}(v_{i}) =\beta_{i} + \alpha\sum\limits_{v_{j} \mid v_{i} \in {N_{G^{T}}(v_{j})}} \frac{C_{pgr}^{So}(v_{j})}{|{N_{G^{T}}(v_{j})}|}   $$

*β*_*i*_ and *α* are constants that relate to the restart and transition probabilities. The PageRank Source of a node is calculated based on the centrality of a its neighbors in the transposed graphs. Define the diagonal in-degree matrix, *D*^′^, of *G* such that [*D*^′^]_*ii*_=*max*(1,*deg*^−^(*v*_*i*_)). Similar to the equations for deriving the standard PageRank, the Source component can be solved as following:

8 $$ C_{pgr}^{So} = \left(I- \alpha A{D^{\prime}}^{-1}\right)^{-1}\beta   $$

Directed centralities only gives importance to either upstream nodes or downstream ones. To address this issue we define the *Source/Sink PageRank*. The fundamental concept of Source/Sink modeling is to measure the centrality of nodes as both sources and sinks of information. We adapt the Source/Sink concept to the PageRank by calculating Source and Sink Centrality values individually and summing them:

9 $$ C_{pgr}^{SS}(v) = C_{pgr}^{So}(v) + C_{pgr}^{Si}(v)   $$

The above definition has no limitation of using different constant parameters for $C_{pgr}^{So}$ and $C_{pgr}^{Si}$, this study uses the same values of *α* and *β* for both components.

*Katz Centrality* is another spectral centrality model where the importance of a node is calculated relative to the sum of centrality of its neighbors. Formally:

10 $$ C_{ktz}(v_{i}) =\beta_{i} + \alpha\sum\limits_{v_{j} \in {N_{G}(v_{i})}} C_{ktz}(v_{j})   $$

In the above formula, *β* is a constant factor and *α* is dampening factor. The convergence of the Formula 10 depends on the largest eigenvalue of the adjacency matrix. In particular, *α*<1/*λ*_1_ is a sufficient condition for convergence, with *λ*_1_ being the largest positive eigenvalue of the adjacency matrix. Rearranging Formula 10 gives a closed form solution of Katz centrality. Formally:

11 $$ C_{ktz} = \left(I - \alpha A\right)^{-1}\beta   $$

where *C*_*ktz*_ is the vector of centrality values. Katz centrality is closely related to the formulations of Cdist and NetGSA for pathway enrichment analysis [16, 18, 24]. Throughout this document, Katz centrality refers to the directed graph. Although Katz centrality is well defined on undirected graphs we will not analyze this model on the dataset of this study since it would impose a small global *α*. We define *Source Katz* ($C_{ktz}^{So}$) component as the standard Katz centrality of a directed graph.

12 $$ C_{ktz}^{So}(v) := C_{ktz}(v)   $$

Next, we define the *Sink Katz*$\left (C_{ktz}^{Si}\right)$ component as the Katz centrality of the transposed graph. In this context, the centrality of a node relates to the centrality of its neighbors in the transposed graph. Formally:

13 $$ \begin{aligned} C_{ktz}^{Si}(v_{i}) & :=\beta_{i} + \alpha \sum\limits_{v_{j} \in {N_{G}^{T}(v_{i})}} C_{ktz}^{Si}(v_{j}) \\ C_{ktz}^{Si} & = \left(I -\alpha A^{T}\right)^{-1}\beta \end{aligned}  $$

In a similar fashion to Source/Sink PageRank. we define the *Source/Sink Katz* as the direct summation of the two individual Source and Sink components. Katz Source/Sink Centrality is then defined as:

14 $$ C_{ktz}^{SS}(v) = C_{ktz}^{So}(v) + C_{ktz}^{Si}(v)   $$

Although the above definition has no limitation of using different constant parameters for individual Source and Sink centralities, this study uses the same values of *α* and *β* for both components. Also, it can be shown that Source and Sink components have the same convergence criteria when using the same *β* and *α*.

*Laplacian* graph influence measures are a family of models that capture the amount of effect a node has on the other nodes. These measures are the core of the heat diffusion kernels of graphs as they relate to the Laplacians of the graph, *D*−*A* [32–35]. Graph Laplacians are generally defined for undirected graphs [32, 35]. There are modifications for directed graphs either on strongly connected graphs or directed acyclic graphs [34, 36]. In this study, we use a specific version for directed graphs that is related to the model used by Shojaie and Michailidis for pathway enrichment analysis (proof of model equivalence in the Appendix)[19]. Though this model is not discussed in the literature with any specific names, to the best of our knowledge, we will refer to it as *Laplacian Centrality*, for the lack of a better terminology. The Laplacian model in this study indicates that the centrality of a node relates to the average centrality of its neighbors. Formally:

15 $$ C_{lap}(v_{i}) =\beta_{i} + \alpha\sum\limits_{v_{j} \in {N_{G}(v_{i})}} \frac{C_{lap}(v_{j})}{|N(v_{i})|}   $$

By expressing the above formulation in matrix format and rearranging for the vector of centralities we get

16 $$ C_{lap} := \left(I- \alpha{D}^{-1}A\right)^{-1} \beta   $$

We define the *Laplacian Source* component as the Laplacian centrality of the directed graph:

17 $$ C_{lap}^{So} := C_{lap}   $$

Similar to the other measures, we define the *Laplacian Sink* component is the Laplacian centrality of the transposed graph:

18 $$ C_{lap}^{Si} := \left(I- \alpha{D^{\prime}}^{-1}A^{T}\right)^{-1} \beta   $$

The *Source/Sink Laplacian* is then defined as the sum of the two components:

19 $$ C_{lap}^{SS} := C_{lap}^{So} + C_{lap}^{Si}   $$

The *Undirected Laplacian* is trivial for the connected components of the graphs since all members of a component can have any equal value for centrality. Therefore, the Laplacian model defined in this report will be only used in directed formats.

The definition of Source/Sink models can be generalized into a format where the contribution of the Source and the Sink components to the total centrality value are weighted. Interested readers may refer to [23, 24] for examples of the weighted definitions– where we have shown that equal weights (as assumed in this article) can generate a SSC value that is most different the individual Source and Sink components.

### Background pathways and genes

#### Human pathways and cancer genes

Human pathways from Kyoto Encyclopedia of Genes and Genomes (KEGG) were retrieved (n = 330, April 2019). We pre-processed pathways and excluded the entries that exhibited 1- more than 1000 nodes and more than 4000 interactions (n = 2) 2- less or equal to 20 nodes or 20 edges (*n* = 86) 3- largest eigenvalues larger than 10 (*n* = 16). In addition, pathways with a single unique value for any of the centrality measures (e.g. all degrees being 10) were excluded from the analysis, resulting in 216 pathways passing the initial quality criteria.

Human cancer genes were retrieved from relevant classifications of Broad Institute’s MSigDB: *Oncogenes*, *Tumor Suppressors*, and *Translocated cancer genes* (*n* = 417, June 2018) [37]. Cancer Gene Census from Sanger Institute was used as an additional reference list for cancer-related genes (*n*= 719, June 2018) [38].

Pathways with 5 or less cancer associated genes were excluded from analysis for consistency of *p*-value calculations (*n* = 61). The final set of pathways contained 157 entries. The pathways were retrieved and analyzed using R-packages “KEGGGraph” and “Pathview” [39, 40].

#### Mouse pathways and lethal genes

Mouse pathways were retrieved from KEGG databased (*n*= 326, April 2019). We used the same quality criteria (edges, nodes, and eigenvalue limits), resulting in 219 pathways. Mouse lethal genes were retrieved from International Mouse Phenotyping Consortium (IMPC) through its online portal (*n* = 1053, June 2018) [41]. The IMPC’s gene family classifications of the related genes were *Lethal*, *Viable*, and *Sub-viable*.

Pathways with 5 or less Lethal genes were excluded from analysis for consistency of *p*-value calculations. The final set of pathways contained 123 entries.

### Experimental methods and analyses

We examine different formats of each centrality model through three aspects. 1– The linear relationship between the centrality scores and the percentage of the genes that are biologically important. 2– The distribution of centrality scores of important genes and other genes (normal). 3– The mean difference between the centrality scores of important genes versus normal genes for each pathway. Since the subjects of study are multiple pathways, rather than a single global graph, normalization and ranking procedures were used to create a unified framework.

This study uses *β*=0.15·*𝟙*_*n*×1_, and *α*=0.85 for different formats of PageRank, as previously recommended in the original PageRank paper [31]. For all Katz centrality formats, the parameter setting was *α*=0.1, *β*=*𝟙*_*n*×1_. Katz models parameters are restricted to be smaller than one over the largest eigenvalue of a graph [24], and thus, we chose the aforementioned parameter to allow for analysis of a reasonable number of pathways. We did not analyze for Undirected Katz because of limitation of the largest eigenvalues. For all Laplacian centrality formats, the parameters were *β*=*𝟙*_*n*×1_, and *α*=0.85. The Laplacian model parameters were chosen to be consistent with PageRank parameters.

#### Regression analysis

Our regression analysis pipeline initially ranks the node of each pathway using one centrality measure at a time. The pipeline then aligns the centrality ranks of within each pathway through 100 quantiles. The 100th quantile indicates most central genes in a pathway and 1st quantile indicates the lowest importance. Formally, let *C*_*a*,*j*_(*v*_*i*_) denote the centrality of a node *v*_*i*_ in pathway *j* using model *a*. The quantile ranking of a node *i*, *Q*_*j*_(*v*_*i*_), is then defined as:

20 $$ Q_{j}(v_{i}) = \left[ 100\times \frac{C_{a,j}(v_{i})}{|V_{j}|} \right]   $$

In the above formula, *V*_*j*_ is the set of nodes in pathway *j*. The quantile ranking allows to compare the centrality rankings among all pathways because different pathways have different number of nodes by assigning the most central node in the highest quantile score. To investigate the relationship between a priori importance of a gene and its centrality, the proportion of important genes were calculated on each quantile across all pathways, with the important genes coming from the cancer genes and lethal genes. The relationship between the centrality and importance were investigated separately based on the gene type, once for cancer genes in human pathways and once for lethal genes in mouse pathways.

Let *Q*_*ij*_ denote the set of genes belonging to *i*-th quantile in pathway *j* — *Q*_*ij*_={*v*∣*v*∈*V*_*j*_,*Q*_*j*_(*v*)=*i*}. Let *R* denote the set of all genes in a class of a priori important genes, i.e., cancer or lethal. We define the percentage of the a priori important genes in *i*-th quantile, *F*_*i*_^*c*^, as:

21 $$ {F_{i}}^{c} = 100 \times \frac{ \sum_{j} \left|\left\{v \mid v \in R \cap Q_{ij} \right\}\right|}{ \sum_{j} \left|\left\{v \mid v \in Q_{ij} \right\}\right|}   $$

Although some genes were occurring in multiple pathways, each occurrence was treated as an unique gene because the purpose was to evaluate the centrality with respect to pathways. *F*_*i*_^*c*^ was then tested against the level of quantile for assessing linear relationships. In the below formula, *i* indicates the index value of a quantile group, e.g. 1 for the 1st quantile and 10 for the 10th quantile. Let *a*_1_ and *a*_0_ be the coefficients of the linear regression. Formally:

22 $$ {F_{i}}^{c} = a_{1}\cdot i + a_{0}   $$

For each centrality measure the above linear regression was fitted and the adjusted r-squared (coefficient of determination) were evaluated. The above procedures were applied to lethal mouse genes and human cancer genes separately in their respective annotated and pre-processed pathways.

#### Comparison of cumulative densities

To compare the distribution of centrality values from a global perspective, the centrality scores were normalized within each pathway using the following formula:

23 $$ N_{a,j}(v_{i}) = \frac{C_{a,j}(v_{i}) - \mu_{a,j}}{\sigma_{a,j}}   $$

where *μ*_*a*,*j*_ and *σ*_*a*,*j*_ are the mean and standard deviation of centrality scores of pathway *j* using method *a*. Accordingly, *N*_*a*,*j*_(*v*_*i*_) is the normalized centrality score of node *v*_*i*_ in pathway *j*, using the centrality method *a*. The normalized score for all pathways were placed in 100 quantiles. The distribution of quantile scores for the types of genes “Important” and “Others” were compared by Kolmogorov-Smirnov (KS) test on cumulative distribution function (CDF) of important and normal genes. The *p*-values were calculated based on the alternative hypothesis of the CDF of the important genes lying below that of the normal. In this test, the CDF of all genes combined would follow a straight line. The described procedure was ran separately for mouse lethal genes and human cancer genes on their respective pre-processed pathways.

#### Within pathways two-Sample testing

For each pathway, the difference of the mean raw centrality values between a priori important genes and other genes were evaluated using Welch’s t-test. Formally:

24 $$ \begin{aligned} t ~ & = \frac{~~~ \hat{\mu}_{a,c} - \hat{\mu}_{a,n}}{\sqrt{\frac{s_{a,c}^{2}}{N_{c}}+\frac{s_{a,n}^{2}}{N_{n}}}} \\ H_{0} &: ~~~ \mu_{a,c} = \mu_{a,n} \\ H_{A} &: ~~~ \mu_{a,c} > \mu_{a,n} \end{aligned}  $$

where $\hat {\mu }_{a,c}$ and $\hat {\mu }_{a,n}$ are the estimated means of centrality values for cancer and normal genes by model *a*. Similarly, $s_{a,c}^{2}$ and $s_{a,n}^{2}$ are the variance estimates of the centrality scores of important and normal genes, using model *a*. *N*_*c*_ and *N*_*n*_ denote the sample size of important genes and normal genes. *H*_0_ is the null hypothesis of important and normal genes having the same mean. *H*_*A*_ is the alternative hypothesis where the important genes have a higher mean.

Since the underlying distribution of the centrality values is unknown, we also used Wilcox non-parametric test to evaluate the null hypothesis of important and non-important genes having the same mean. Wilcox test ranks individual observations and evaluates the difference between the sum of the ranking in two classes of the hypothesis. While Wilcox test is the more appropriate choice for testing this study’s hypotheses, we present both parametric (Welch) and non-parametric (Wilcox) two-sample tests for comparison.

For each centrality model, the *p*-values from Formula 24 and Wilcox test were calculated across all pathways. Because of the large number of pathways, multiple-hypothesis testing corrected criterion was used to determine significant *p*-values. In particular, Benjamini-Hochberg False Discovery Rate was applied to all calculated *p*-values for each centrality method to control type-I error at %5 (*FDR*<0.05) [42]. The same procedure was applied to both parametric and non-parametric approaches. The sets of significant pathways for each centrality model were contrasted against each other. The described procedure in this subsection was applied to human cancer genes and mouse lethal genes in separate analyses.

## Results

### Pathway centrality of human cancer genes

**Fig. 1:**
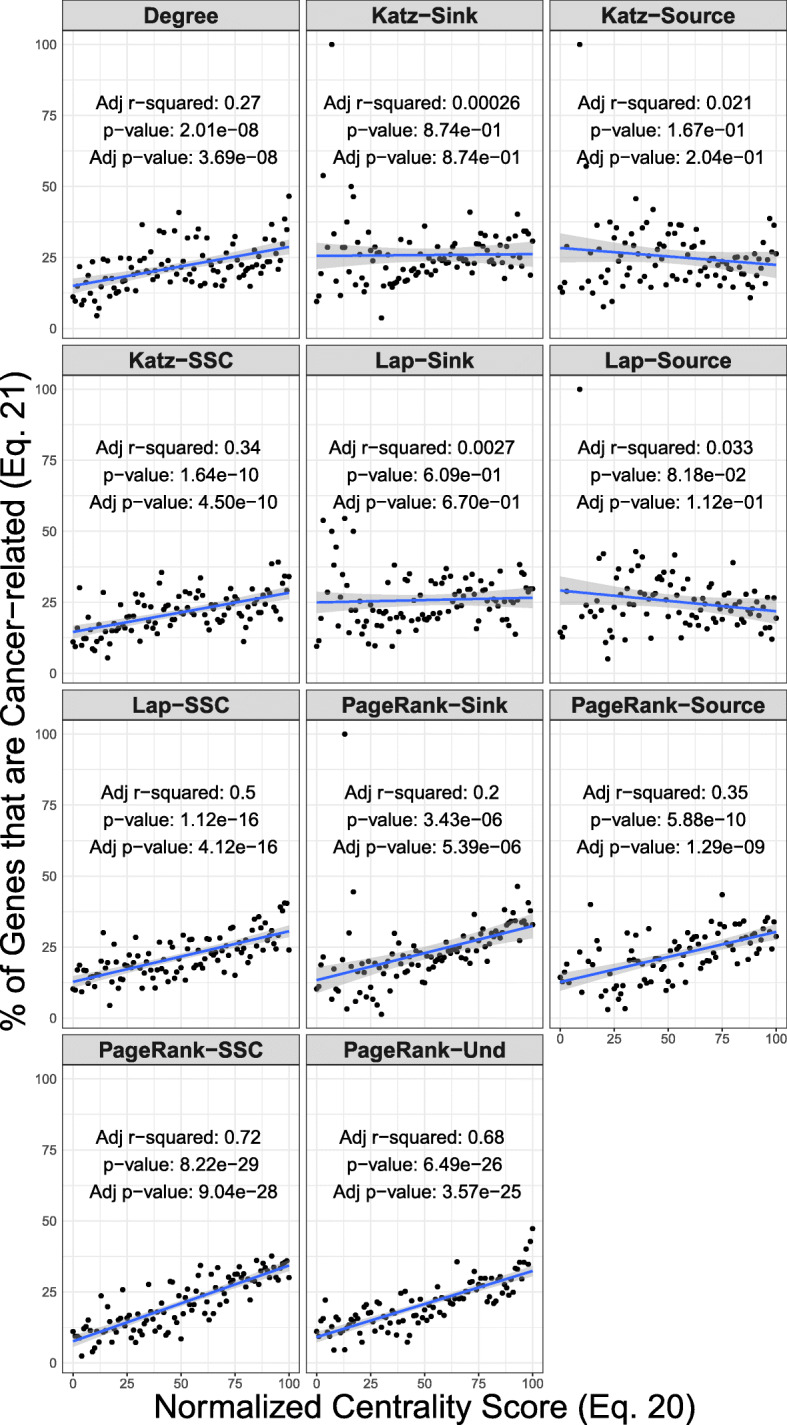
Linear regression fit of the quantile-normalized centrality scores (Eq. 20) and the percentage human pathway genes that are cancer-related (Eq. 21). The Source/Sink extension of the centrality models show higher slope and adjusted coefficient of determination (Adjusted r-squared) in comparison to the standard variations of the centrality models (Table 1)

The regression analysis shows an evident increase in the percentage of cancer genes with the increased centrality scores from Degree, Katz Source/Sink, Laplacian Source/Sink, and all PageRank formats (Fig. 1, Table 1). For all of the other models, the analysis fails to identify any linear relationship between the centrality scores and the percentage.

**Table 1:** Linear regression fit of the quantile-normalized centrality scores (Eq. 20) and the percentage human pathway genes that are cancer-related

Centrality	term	estimate	std.error	statistic	*p*.value
Degree	(Intercept)	1.50e+01	1.30e+00	1.15e+01	4.98e-20
Degree	Coefficient	1.37e-01	2.24e-02	6.11e+00	2.01e-08
Katz-Sink	(Intercept)	2.55e+01	2.31e+00	1.11e+01	8.26e-19
Katz-Sink	Coefficient	6.23e-03	3.93e-02	1.58e-01	8.74e-01
Katz-Source	(Intercept)	2.84e+01	2.57e+00	1.10e+01	1.49e-18
Katz-Source	Coefficient	-5.97e-02	4.28e-02	-1.39e+00	1.67e-01
Katz-SSC	(Intercept)	1.45e+01	1.12e+00	1.30e+01	4.23e-23
Katz-SSC	Coefficient	1.38e-01	1.93e-02	7.13e+00	1.64e-10
Lap-Sink	(Intercept)	2.49e+01	1.89e+00	1.32e+01	1.92e-23
Lap-Sink	Coefficient	1.65e-02	3.23e-02	5.12e-01	6.09e-01
Lap-Source	(Intercept)	2.91e+01	2.50e+00	1.17e+01	8.94e-20
Lap-Source	Coefficient	-7.29e-02	4.14e-02	-1.76e+00	8.18e-02
Lap-SSC	(Intercept)	1.27e+01	1.03e+00	1.24e+01	8.89e-22
Lap-SSC	Coefficient	1.78e-01	1.78e-02	9.99e+00	1.12e-16
PageRank-Sink	(Intercept)	1.33e+01	2.28e+00	5.85e+00	7.09e-08
PageRank-Sink	Coefficient	1.91e-01	3.86e-02	4.93e+00	3.43e-06
PageRank-Source	(Intercept)	1.27e+01	1.54e+00	8.23e+00	1.37e-12
PageRank-Source	Coefficient	1.77e-01	2.55e-02	6.94e+00	5.88e-10
PageRank-SSC	(Intercept)	7.58e+00	9.78e-01	7.76e+00	7.88e-12
PageRank-SSC	Coefficient	2.67e-01	1.69e-02	1.58e+01	8.22e-29
PageRank-Und	(Intercept)	9.06e+00	9.39e-01	9.65e+00	6.36e-16
PageRank-Und	Coefficient	2.33e-01	1.62e-02	1.43e+01	6.49e-26

For the Degree centrality, as shown in Fig. 1, the higher values of quantile scores correspond to higher percentages points of cancer genes, and low quantile scores exhibit lower percentage of cancer genes. The analysis supports this observation by showing a linear relationship between the scores and the percentage of genes that are cancer-related with an adjusted r-squared (Adj *r*^2^) of 0.27. The regression analysis shows a statistically significant positive coefficient of 1.37×10^−1^ for the quantile scores (Adj *p*-value = 3.96×10^−8^, Table 1).

The standard directed variation of Katz centrality (the Source component) fails to identify an evidence (Adj *p*-value = 0.167) for linear relationship (Fig. 1), and the linear regression model accounts for an insignificant fraction of the variance (Adj *r*^2^ = 0.021). Similarly, the Katz Sink Component produces an Adj *r*^2^ = 0.00026 and Adj *p*-value = 0.874. In contrast, the combined value of the two components, Source/Sink Katz, shows that the linear relationship explains a statistically significant portion of the variance (Adj *r*^2^ = 0.34). In this case, the regression analysis shows a statistically significant positive coefficient of 1.38×10^−1^ for the quantile scores (Adj *p*-value = 4.50×10^−10^, Table 1).

**Fig. 2:**
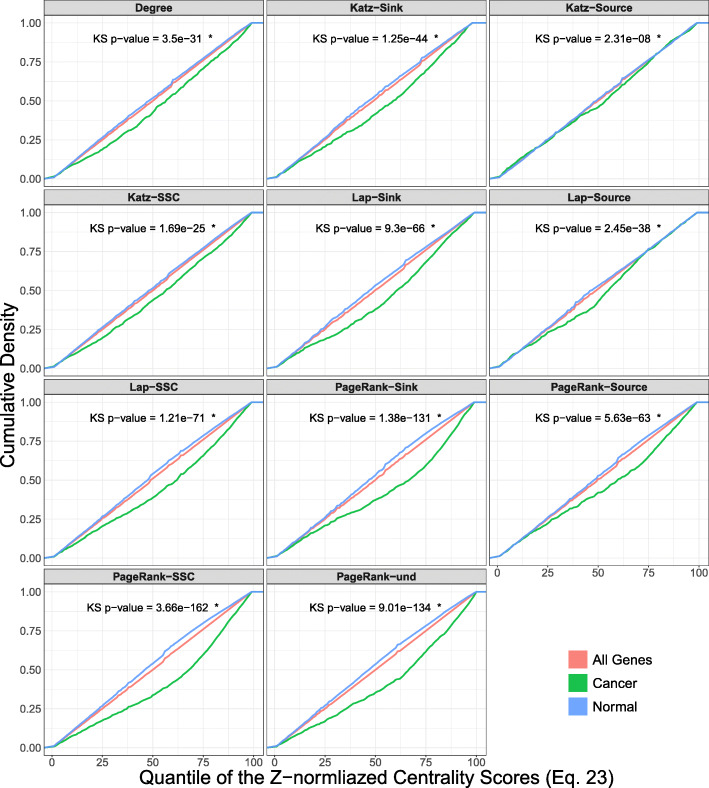
Comparison of the cumulative density between cancer-related genes and normal genes. The data points represent the quantile-scores calculated based on normalized centrality (Formula 23) across all pathways. Each panel includes the *p*-value of Kolmogorov-Smirnov test for the hypothesis of the CDF of cancer genes being below that of the normal genes. The panels show that cancer genes tend to have higher centrality values according to all of the models. This indicates the individual values of source and sink components for capturing the topological importance of cancer genes. Asterisk marks denote the *p*-values generate by the KS test method in R

For different formats of Katz centrality, comparison of cumulative distribution function (CDF) between the cancer genes and normal genes shows that the CDF of cancer genes lies below that of the normal genes (Fig. 2). Although all of the differences are statistically significant, amount of differences depends on the specific variation of centrality. For example, more than 65% of the cancer genes have a normalized quantile score greater than 50 when measuring centrality using Katz Source/Sink method. The pathway-by-pathway two-sample tests also show that each variation of Katz centrality is able to detect a number of pathways to have a higher mean of centrality for cancer genes (Tables 2 and 3). The number of identified pathways in the non-parametric model are higher in comparison to a regular t-test. For example, the two-sample t-test detects five pathways with higher centrality of cancer genes using Katz Source/Sink, while the Wilcox rank-sum test identifies 13 pathways (*FDR*<0.05).

**Table 2:** Pathways identified with higher mean centrality for cancer genes by t-test

	Deg	Katz			Lap			Pgr			
	Deg	Sink	So	SSC	Sink	So	SSC	So	Si	SSC	Und
Degree	5	0	2	4	0	2	1	0	1	0	2
Katz-Sink		6	0	1	5	0	1	2	0	2	1
Katz-Source			2	2	0	2	1	0	1	0	0
Katz-SSC				5	1	2	1	0	1	0	1
Lap-Sink					17	0	4	3	0	3	1
Lap-Source						8	2	0	1	0	0
Lap-SSC							15	2	1	2	1
Pgr-Sink								5	1	3	1
Pgr-Source									2	1	1
Pgr-SSC										5	2
Pgr-Und											7

**Table 3:** Pathways identified with higher mean centrality for cancer genes by Wilcox test

	Deg	Katz			Lap			Pgr			
	Deg	Sink	So	SSC	Sink	So	SSC	So	Si	SSC	Und
Degree	9	1	4	9	1	2	2	1	3	3	9
Katz-Sink		13	1	2	10	1	3	12	2	10	7
Katz-Source			9	5	1	5	2	4	6	4	8
Katz-SSC				13	2	2	2	3	4	5	11
Lap-Sink					20	2	6	13	2	11	6
Lap-Source						8	3	4	7	4	5
Lap-SSC							15	7	5	6	3
Pgr-Sink								25	8	20	13
Pgr-Source									16	12	9
Pgr-SSC										31	15
Pgr-Und											29

Comparison of different PageRank centrality formats also shows that the SSC framework produces a better description of the pathway organizations compared to the individual Source and Sink components. The centrality values of individual Source (Adj *p*-value = 1.29×10^−9^ and Adj *r*^2^=0.35) and Sink (Adj *p*-value = 5.39×10^−6^, and Adj *r*^2^=0.20) components of PageRank have linear relationship with the percentage of cancer genes. The combination of the two components as in the Source/Sink PageRank produces a more descriptive and stronger linear relationship in term of both the adjusted r-squared and the regression coefficient (Adj *p*-value = 9.04×10^−28^ and Adj *r*^2^=0.72). Undirected PageRank also provides a stronger linear relationship in comparison to the individual Source and Sink components (Adj *p*-value = 3.57×10^−25^ and Adj *r*^2^=0.68).

For different formats of PageRank, comparison of the CDF between the cancer genes and normal genes shows that the CDF of cancer genes lies below that of the normal genes (Fig. 2). Although all of the differences are statistically significant, amount of differences depends on the specific variation of centrality. This indicates that each format of PageRank shows higher values of centrality for cancer genes in the distribution of the scores, even though the distinction may not be apparent according to the linear regression model. For example, more than 70% of the cancer genes have a normalized quantile score greater than 50 when measuring centrality using PageRank Source/Sink method. The pathway-by-pathway two-sample tests also show that each variation of PageRank centrality is able to detect pathways with a higher mean of centrality for cancer genes (Tables 2 and 3). In this case, the number of identified pathways in the non-parametric model are higher in comparison to a regular t-test. For example, the two-sample t-test detects five pathways with higher centrality of cancer genes using PageRank Source/Sink, while the Wilcox rank-sum test identifies 31 pathways (*FDR*<0.05).

The Laplacian model also detects a linear relationship only when considering the Source/Sink formatting (Adj *p*-value = 4.12×10^−16^ and Adj *r*^2^=0.50). For different formats of Laplacian, comparison of the CDF between the cancer genes and normal genes shows that the CDF of cancer genes lies below that of the normal genes (Fig. 2). The pathway-by-pathway two-sample tests also show that each variation of Laplacian centrality is able to detect a number of pathways to have a higher mean of centrality for cancer genes (Tables 2, and 3). In this case, the number of identified pathways in the non-parametric model are similar to a regular t-test. For example, the two-sample t-test detects fifteen pathways with higher centrality of cancer genes using Laplacian Source/Sink, the same number as the Wilcox rank-sum (*FDR*<0.05).

**Fig. 3:**
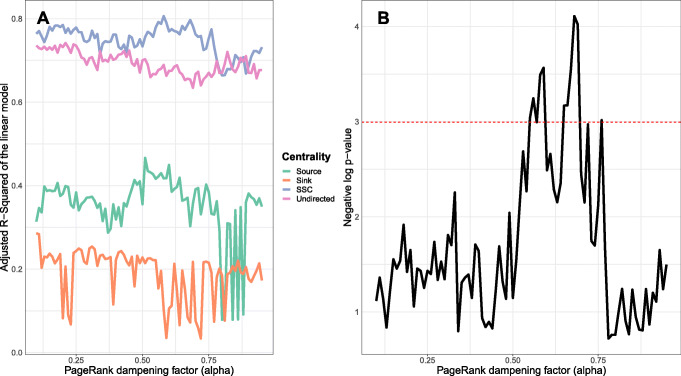
Sensitivity analysis of PageRank variations in the linear regression analysis with respect to the *α* parameter. Panel A shows the adjusted r-squared of the linear fit per *α*(Formula 22). Panel B displays the negative log *p*-value of the difference between the correlation coefficients of SSC PageRank versus undirected PageRank. The red line in panel B denotes the significance threshold of *p*-value=0.05

We wondered whether the differences between the SSC framework and other variations were sensitive to the choice of model-specific parameters. To address this question, we repeated the regression analysis for the four variations of PageRank across the values of *α*∈[0.1,0.9] with 0.01 increments (across all the pathways, n = 157). We then compared the adjusted r-squared of the linear regression models according to Formula 22 (Fig. 3). We used Fisher’s Z-transformation of correlation coefficients of the linear fits to measure the statistical difference between SSC and undirected PageRank across the range of *α*. The results are suggestive that PageRank SSC produces the highest adjusted r-squared among all the variations for the most of the search range, and as well as the undirected model for some part of the range. In particular, SSC produces the highest r-squared for any variation of PageRank at any alpha at *α*=0.58.

**Fig. 4:**
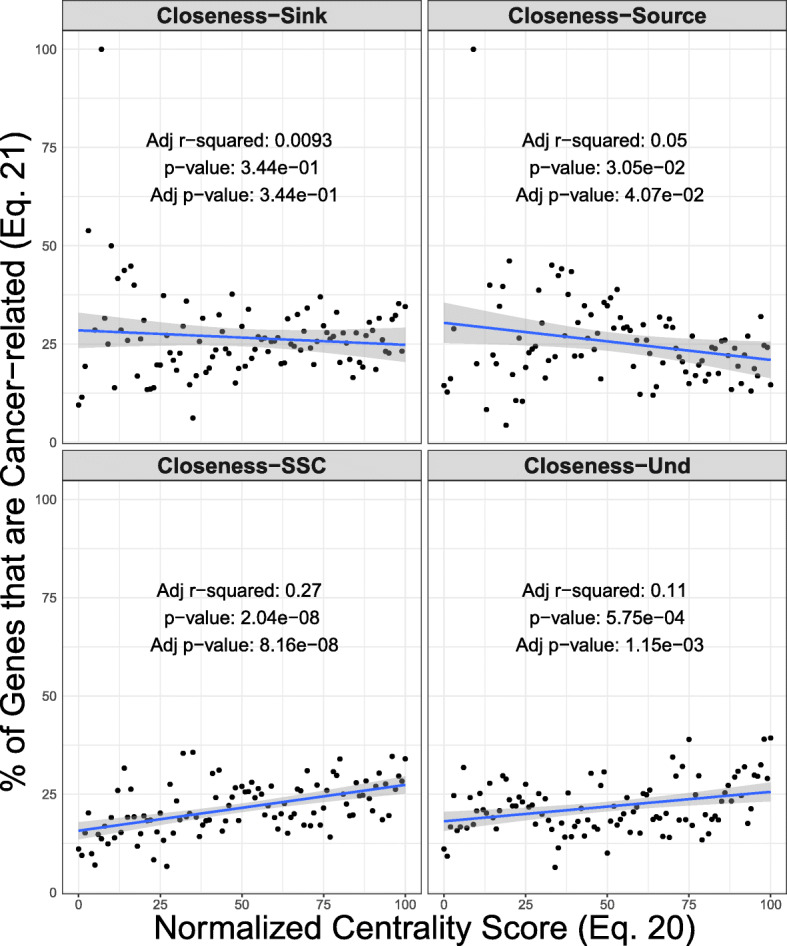
Linear regression fit of the quantile-normalized centrality scores (Eq. 20) and the percentage genes that are cancer-related (Eq. 21). The Source/Sink extension of the centrality models show higher slope and adjusted coefficient of determination (Adjusted r-squared) in comparison to the standard variations of the centrality models

We also tested the linear regression framework for closeness centrality, as an example of a centrality method that is not spectral (details in Appendix). Our analysis shows that Source/Sink closeness centrality is able to identify a linear relationship between quantile-scores and percentage points of cancer genes (Adj *r*^2^=0.27) while individual Source and Sink components fail to identify. Also, undirected closeness centrality shows a lower coefficient of determination (Adj *r*^2^=0.11) in comparison to the SSC variation.

### Pathway centrality of mouse lethal genes

**Fig. 5:**
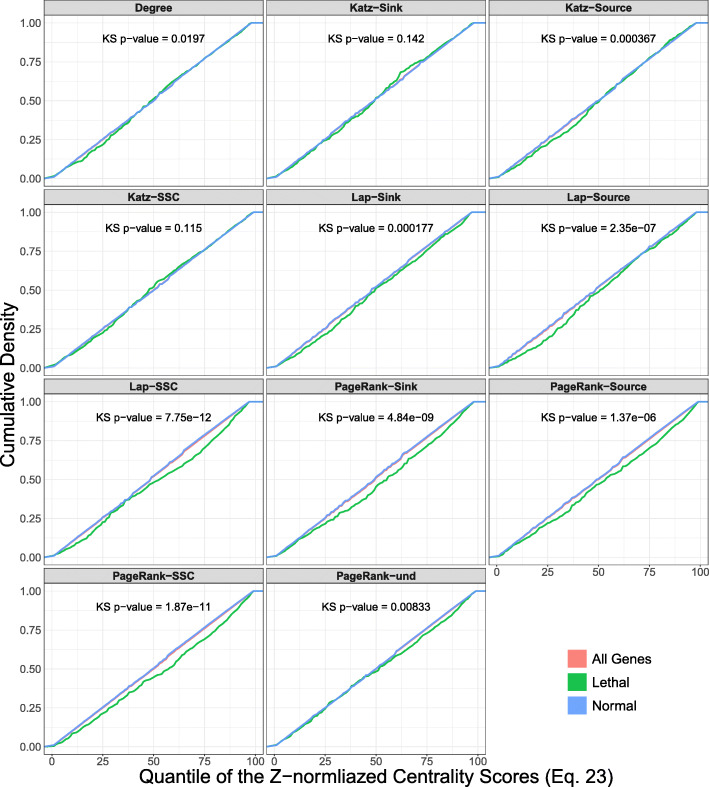
Linear regression fit of the quantile-normalized centrality scores (Eq. 20) and the percentage mouse pathway genes that are lethal (Eq. 21). The Source/Sink extension of the centrality models show higher slope and adjusted coefficient of determination (Adjusted r-squared) in comparison to the standard variations of the centrality models (Table 4)

The regression analysis shows an evident increase in the percentage of the genes that are lethal in mouse pathways with the increased centrality scores from PageRank Source/Sink, Laplacian Source/Sink, and undirected PageRank (Fig. 5). For all of the other models, the analysis fails to identify any linear relationship between the centrality scores and the percentages.

The Source PageRank centrality fails to identify any evidence (Adj *p*-value = 0.649) for linear relationship (Fig. 5), and the linear regression model accounts for an insignificant fraction of the variance (Adj *r*^2^ = 0.0022), similar to values for the PageRank Sink Component (Adj *r*^2^ = 0.004 and Adj *p*-value = 0.55). In contrast, the combined value of the two components, Source/Sink PageRank, shows that the linear relationship explains a statistically significant portion of the variance (Adj *r*^2^ = 0.21). In this case, the regression analysis shows a statistically significant positive regression coefficient of 5.24×10^−2^ (Table 4).

**Table 4:** Linear regression fit of the quantile-normalized centrality scores (Eq. 20) and the percentage of mouse pathway genes that are lethal

Centrality	term	estimate	std.error	statistic	p.value
Degree	(Intercept)	8.08e+00	5.86e-01	1.38e+01	1.19e-24
Degree	Coefficient	1.14e-02	1.01e-02	1.13e+00	2.62e-01
Katz-Sink	(Intercept)	1.08e+01	1.07e+00	1.01e+01	9.91e-17
Katz-Sink	Coefficient	-2.27e-02	1.79e-02	-1.27e+00	2.08e-01
Katz-Source	(Intercept)	1.32e+01	1.09e+00	1.21e+01	6.70e-21
Katz-Source	Coefficient	-5.63e-02	1.83e-02	-3.08e+00	2.69e-03
Katz-Source-Sink	(Intercept)	8.47e+00	6.78e-01	1.25e+01	4.66e-22
Katz-Source-Sink	Coefficient	6.40e-03	1.17e-02	5.46e-01	5.86e-01
Lap-Sink	(Intercept)	1.03e+01	1.15e+00	8.89e+00	4.62e-14
Lap-Sink	Coefficient	-1.06e-02	1.94e-02	-5.48e-01	5.85e-01
Lap-Source	(Intercept)	1.30e+01	1.15e+00	1.13e+01	3.82e-19
Lap-Source	Coefficient	-4.50e-02	1.93e-02	-2.33e+00	2.20e-02
Lap-SSC	(Intercept)	5.51e+00	7.05e-01	7.82e+00	5.70e-12
Lap-SSC	Coefficient	6.65e-02	1.22e-02	5.46e+00	3.54e-07
PageRank-Sink	(Intercept)	8.72e+00	1.05e+00	8.28e+00	1.17e-12
PageRank-Sink	Coefficient	1.04e-02	1.74e-02	6.01e-01	5.50e-01
PageRank-Source	(Intercept)	9.15e+00	1.15e+00	7.93e+00	4.85e-12
PageRank-Source	Coefficient	8.83e-03	1.94e-02	4.56e-01	6.49e-01
PageRank-SSC	(Intercept)	5.92e+00	5.84e-01	1.01e+01	5.26e-17
PageRank-SSC	Coefficient	5.24e-02	1.01e-02	5.20e+00	1.09e-06
PageRank-Und	(Intercept)	7.12e+00	5.26e-01	1.35e+01	2.81e-24
PageRank-Und	Coefficient	2.67e-02	9.08e-03	2.94e+00	4.03e-03

**Fig. 6:**
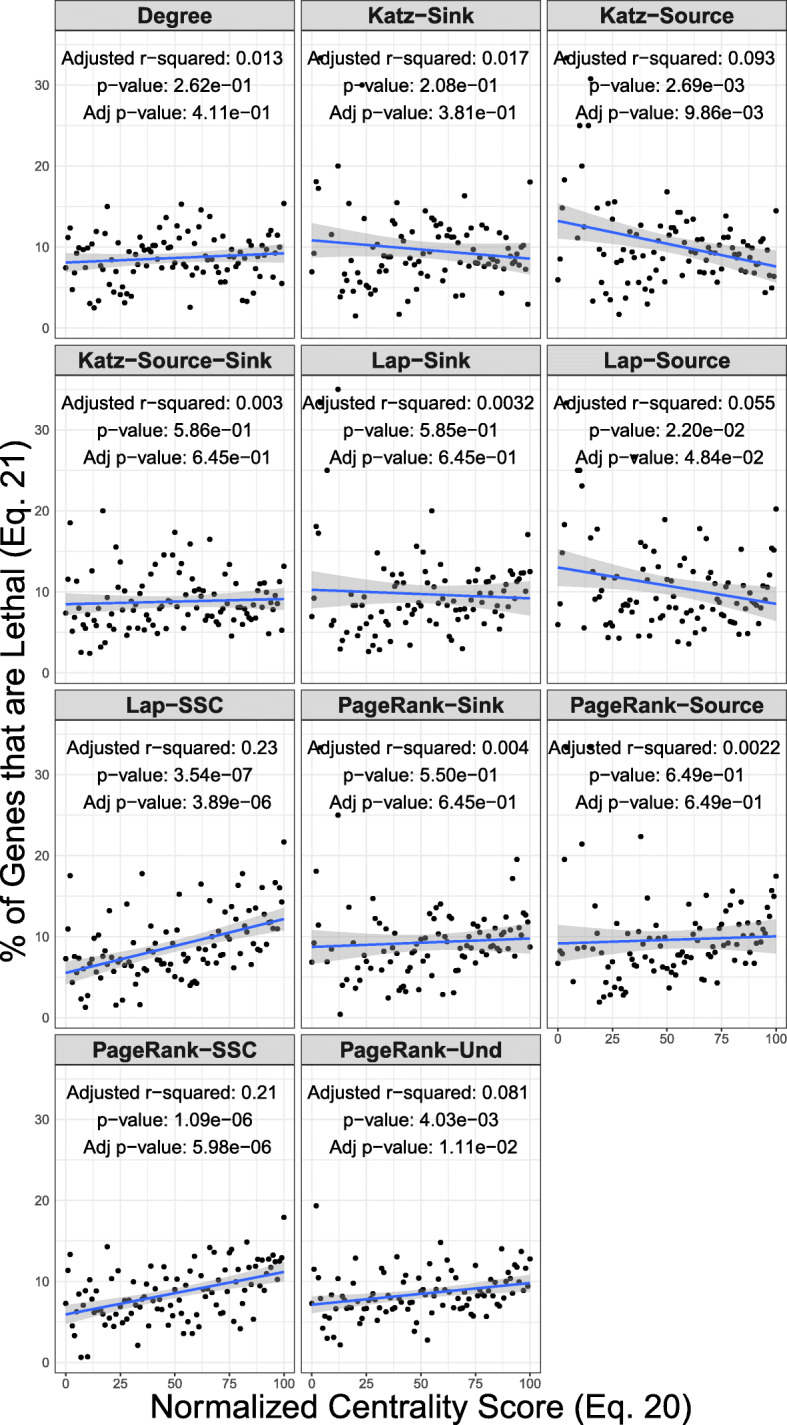
Comparison of the cumulative density between lethal genes and normal (non-lethal) genes. The data points represent the quantile-scores calculated based on normalized centrality (Formula 23) across all pathways. Each panel includes the *p*-value of Kolmogorov-Smirnov test for the hypothesis of the CDF of lethal genes being below that of the normal genes. The panels show that lethal genes tend to have higher centrality values according to some of the models, including Source/Sink PageRank and Source/Sink Laplacian

For different formats of all centrality models, comparison of CDF between the cancer genes and normal genes shows that the CDF of lethal genes lies below that of the normal genes (Fig. 6). The two-sample tests shows statistical power in detecting pathways with higher centrality of lethal genes using a less conservative FDR threshold (Tables 5, and 6). The pathway-by-pathway two-sample tests also show that each variation of Laplacian centrality and some formats of PageRank are able to detect a number of pathways to have a higher mean of centrality for cancer genes. For Laplacian centrality, the number of identified pathways in the non-parametric model is similar in comparison to a regular t-test.

**Table 5:** Pathways identified with higher mean centrality for mouse lethal genes by Wilcox test (*FDR*<0.25)

	Lap			Pgr		
	Sink	So	SSC	Sink	So	SSC
Lap-Sink	1	0	1	0	1	0
Lap-Source		1	0	0	1	1
Lap-SSC			4	0	2	0
Pgr-Sink				2	0	0
Pgr-Source					6	2
Pgr-SSC						4

**Table 6:** Pathways identified with higher mean centrality for mouse lethal genes by t-test (*FDR*<0.25)

	Lap		Pgr
	Sink	SSC	Sink
Lap-Sink	1	1	0
Lap-SSC		6	0
Pgr-Sink			1

## Discussion

Regression analysis of the topological position of cancer genes in human pathways shows that the graph centrality models can account for the percentage of the known important genes, particularly when formulated in Source/Sink modeling. Individual source or sink components of Katz and Laplacian fail to identify evidence for the linear relationship of centrality with the percentage of the genes that are cancer-related, noting these models have been applied in different pathway analysis methods [15, 16, 18]. In contrast, the SSC format of Katz and Laplacian models exhibit a statistically significant linear relationship between the centrality and the percentage of the genes that are cancer-related (Table 1). This improvement is due to SSC assigning centrality values to nodes that are downstream terminal but topologically important as receivers of information.

Similarly, SSC PageRank shows significant improvement in comparison to the standard directed formats in explaining the topological importance of cancer genes. These observations provide a noteworthy insight as different tools leverage directed formats of PageRank in pathway analysis applications [14, 17]. The higher adjusted *r*^2^ of SSC compared to other standard variations (Fig. 3) may be explained by noting that SSC PageRank is sensitive both to the directionality and the position at the upstream/downstream organization of pathways. Consistently, for every one of the centrality models the adjusted *r*^2^ and the slope of the linear regression coefficient increase when using the Source/Sink framework.

The analysis of lethal genes in mouse pathways provide additional validation for SSC methodology. The association of lethal genes with topological importance in biological networks has been extensively studied in the context of protein-protein interaction networks, namely centrality-lethality rule [28–30]. Our results provide an account for the centrality-lethality rule in the biological pathways, noting that the pattern is statistically significant when leveraging SSC modeling. As evident in Fig. 5, SSC formats of Laplacian and PageRank, and undirected PageRank to some extent provide evidence for the centrality-lethality rule.

Lack of linear relationship in topological importance versus the percentage of biologically important genes may not dismiss the utility of a centrality model. As evident in CDF analysis and two-samples tests, a centrality model may exhibit distinct patterns between cancer (lethal) and non-cancer (non-lethal) genes. This CDF evidence may explain why the combination of the source and sink components is more informative. We believe that the evidence of increasing linear relationship between the topological importance and the percentage of biologically important genes provides a critical insight with respect to the appropriate choice of directed graph modeling in pathways. In particular, our results strongly indicate that the knowledge of the topological importance of downstream nodes is as valuable as that of upstream nodes, and should not be dismissed as irrelevant as assumed by current pathway analysis models [14, 15, 18]. In fact, our results demonstrate a setting wherein the use of SSC and Undirected modeling is superior to the directed formats. In these conditions, the PageRank SSC can provide a better explanation of the linear relationship in comparison to the undirected model for several possible values of *α*.

A limitation of the present study is the requirement of having sufficient information on the underlying network of pathways. Pathway databases can contain several entries with insufficient number of interaction between genes and other bio-molecular entities. In such cases, topological analysis is not feasible and some intermediate steps, such as data-driven prediction of interactions, may be necessary before using any network-based modeling [20]. Another limitation of SSC modeling is the requirement of having information on the directionality of interactions, which can be absent in some pathway datasets.

While SSC modeling is simple and straightforward, any potential application in other centrality methods should be handled with caution. We only focused on spectral centrality models because of their widespread use in biological network analysis and their compatibility to theoretically express the SSC framework. For other centrality types, SSC framework may or may not be the best option. For example, betweenness centrality – which measure importance of a node relative to the number of shortest paths that pass through it – does not produce a meaningful Source Sink variation. If a node *k* falls within a *vu* shortest path in a graph *G*, it also falls within the *uv* shortest path in *G*^*T*^. In another example in the Appendix, we demonstrate the linear regression model of cancer genes in human pathways using SSC modeling in closeness centrality, which is consistent with the presented results.

The presented results are concordant with our hypothesis that accounting for the upstream and downstream organization of pathways provides more biologically relevant assessment of organization of pathways. The presented results also explain the success of Source/Sink modeling in achieving higher sensitivity and biological relevance in the enrichment analysis and functional interpretation of genomic data as was presented in our previous research [24]. When considering the biological context, our results formalize the intuitive observation that the key pathway elements may appear at any stage of the pathways. The presented results also highlight a disadvantage of the directed pathway analysis models that fundamentally assume a higher importance for the upstream pathway elements and neglect the changes/perturbations of downstream elements. The appropriate choice of centrality measures for biological network analyses may vary depending on model assumptions and the underlying data. However, when applicable, our results recommend adapting SSC framework for fully leveraging the underlying structure of the networks.

## Conclusion

This study investigated the explanatory power of different centrality models with respect to a priori important pathway genes. We tested standard and novel centrality models, and presented a novel alternative with a better topological description of the pathways that accounts for the importance of the pathway elements with respect to the upstream and downstream positions. The two case examples in this study were cancer/non-cancer genes in human and lethal/viable genes in mouse. For both groups there exists literature on their positions and importance in their corresponding biological networks.

Regression analysis, subsequent comparison of CDFs, and two-sample tests of the pathways show that spectral importance determines the topological importance of cancer genes. In particular, the SSC modeling results in more distinct and clear separation of the a priori important genes. These results show that using directions while giving importance to terminal nodes in pathways may give higher explanatory power which should be of particular interest to the research in biological networks and pathway analysis.

## Appendix

### Closeness centrality

Closeness centrality describes a model where the importance of each node is calculated as the sum of its shortest distance from all the other nodes. Formally:

25 $$ C_{cls}(v) := \sum_{u \in V(G)}\frac{1}{d(v,u)}   $$

where *d*(*v*,*u*) denotes the length of the shortest paths between *v* and *u*. Similar to the other models, we define *source closeness* as the standard closeness centrality.

26 $$ C_{cls}^{So}(v) := C_{cls}(v)   $$

We define *sink closeness* as:

27 $$ C_{cls}^{Si}(v) := \sum_{u \in V(G)}\frac{1}{d(u,v)}   $$

Subsequently, we defined the Source Sink closeness centrality as:

28 $$ C_{cls}^{SS} := C_{cls}^{So} + C_{cls}^{Si}   $$

We analyzed the linear regression fit of quantile-normalized closeness centrality values versus the percentage of genes that are cancer-related in human pathways. The results show a higher adjusted r-squared for the SSC variation compared to the other models.

### Proofs

For an adjacency matrix of a directed graph, *A*, define the weight normalized matrix *L* using a positive real value *d* as following:

29 $$ L_{ij}(d) = \frac{A_{ij}}{d +\sum_{j=1}^{n} |A_{ij}|}  $$

30 $$ L = {\lim}_{d \to 0} L(d)  $$

Define the influence matrix, *L*^∗^, as the geometric series of L. In the case of undirected graphs, this notion is related to the concept of normalized Laplacian and heat diffusion kernels [32].

31 $$ L^{*} = \sum_{i=0}^{\infty} L^{i}   $$

On the condition of convergence, the above summation can be written as:

32 $$ L^{*} = {\lim}_{d \to 0} (I - L(d))^{-1}  $$

According to Shojaie and Michailidis, choice of *d* as small as 0.01 would produce consistent and stable results. However, to eliminate the need for the parameter *d*, we rewrite an equivalent formulation for the matrix *L* as :

33 $$ L := D^{-1}A  $$

where *D* is the diagonal degree matrix with the same definition as in *D* of PageRank. As noted in [22], for undirected graphs, the solution to the matrix *L* in a matrix geometric series uniquely exist. That is, the matrix *L*^∗^ from Formula 31 is only guaranteed to uniquely exist when we use the symmetric matrix of the undirected graph.

However, the case might be different for directed graphs. Therefore, including a shrinking factor, *α*<1, that ensures the convergence in a geometric summation. We then re-define:

34 $$ L := \alpha D^{-1}A  $$

Using the above Formula, we define the Laplacian centrality of a node as the aggregated influence of a node *i* on all other nodes. This is obtained from Formula 31:

35 $$ \begin{aligned} C_{lap} & = L^{*} \mathbbm{1} \\ & = \left(I- \alpha D^{-1}A\right)^{-1} \mathbbm{1} \end{aligned}  $$

## Data Availability

All datasets were retrieved from public repositories. The code for analysis is available throught the following address: https://github.com/pouryany/CentralityInPathways
